# Adapting the pre-trained convolutional neural networks to improve the anomaly detection and classification in mammographic images

**DOI:** 10.1038/s41598-023-41633-0

**Published:** 2023-09-09

**Authors:** Abeer Saber, Abdelazim G. Hussien, Wael A. Awad, Amena Mahmoud, Alaa Allakany

**Affiliations:** 1 Department of Information Technology, Faculty of Computers and Artificial Intelligence, Damietta University, Damietta, 34517 Egypt; 2 Department of Computer and Information Science, Linköping University, Linköping, Sweden; 3 Faculty of Science, Fayoum University, Faiyum, Egypt; 4 MEU Research Unit, Middle East University, Amman, 11831 Jordan; 5 Department of Computer Science, Faculty of Computers and Artificial Intelligence, Damietta University, Damietta, 34517 Egypt; 6 Department of Computer Science, Faculty of Computers and Information, KafrElSheikh University, Kafr El‑Sheikh, 33511 Egypt

**Keywords:** Computational science, Computer science, Information technology, Software

## Abstract

Mortality from breast cancer (BC) is among the top causes of cancer death in women. BC can be effectively treated when diagnosed early, improving the likelihood that a patient will survive. BC masses and calcification clusters must be identified by mammography in order to prevent disease effects and commence therapy at an early stage. A mammography misinterpretation may result in an unnecessary biopsy of the false-positive results, lowering the patient’s odds of survival. This study intends to improve breast mass detection and identification in order to provide better therapy and reduce mortality risk. A new deep-learning (DL) model based on a combination of transfer-learning (TL) and long short-term memory (LSTM) is proposed in this study to adequately facilitate the automatic detection and diagnosis of the BC suspicious region using the 80–20 method. Since DL designs are modelled to be problem-specific, TL applies the knowledge gained during the solution of one problem to another relevant problem. In the presented model, the learning features from the pre-trained networks such as the squeezeNet and DenseNet are extracted and transferred with the features that have been extracted from the INbreast dataset. To measure the proposed model performance, we selected accuracy, sensitivity, specificity, precision, and area under the ROC curve (AUC) as our metrics of choice. The classification of mammographic data using the suggested model yielded overall accuracy, sensitivity, specificity, precision, and AUC values of 99.236%, 98.8%, 99.1%, 96%, and 0.998, respectively, demonstrating the model’s efficacy in detecting breast tumors.

## Introduction

Cancer is a group of diseases that bring together cells in the body to form lumps called malignant tumors. In an uncontrolled way, these cells expand, and spread throughout neighbouring tissues, pushing out the normal cells. Cancer is considered to be one of the most significant diseases damaging human health, from the past to the present.

**Figure 1:**
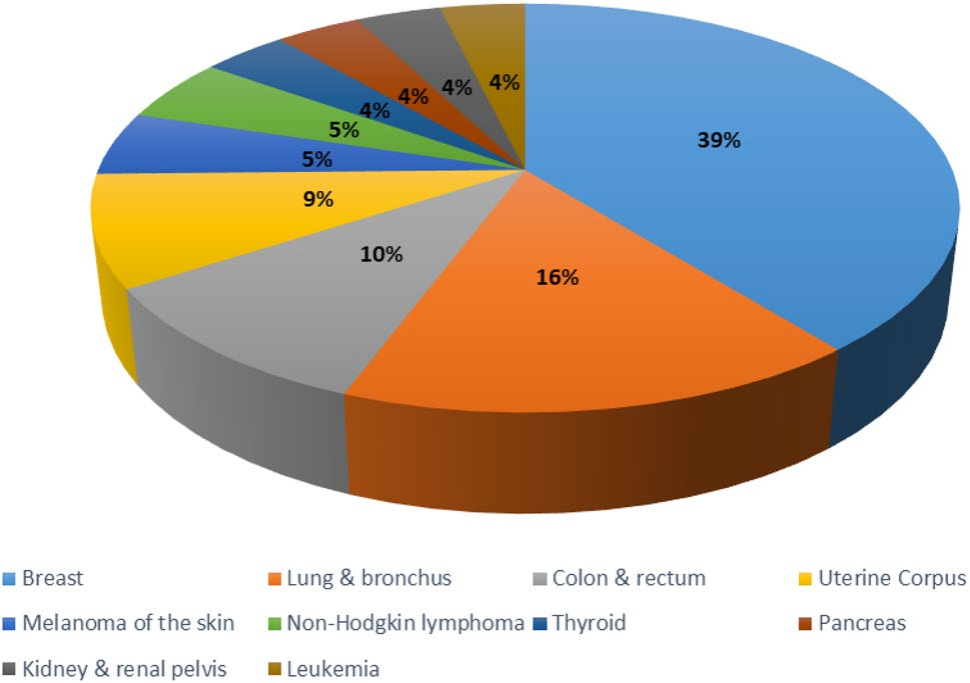
The estimated new cancer cases in 2023.

BC, lung cancer, and colorectal cancer will account for 52% of all new diagnoses in women in 2023, with BC alone accounting for 31% of female cancers. Since the mid-2000s, female BC incidence rates have been slowly increasing by roughly 0.5% per year, owing primarily to diagnoses of localized stage and hormone receptor-positive illness. This trend has been attributed, at least in part, to continued declines in fertility rates and increases in excess body weight, which may also contribute to an increase in uterine corpus cancer incidence of about 1% per year among women aged 50 and older since the mid-2000s and nearly 2% per year in younger women since at least the mid-1990s^1–3^. Figure  1 illustrates the estimated new cancer cases in 2023 for females in the United States.

One of the most important screening techniques for detecting BC is mammography, a special X-ray of a woman’s breast. Compared to mammography, a 3D mammogram is a powerful model. To create a 3D image of the breast, a 3D mammogram uses several breast X-rays. For women who have no signs or symptoms, a 3D mammogram is used to diagnose BC. It may also be used to look at other breast problems, such as breast mass, irritation, and nipple discharge^4^. The daily increase in the number of mammograms increases the radiologist’s workload, resulting in an increase in the misdiagnosis rate. However, regardless of the expertise of the clinicians studying mammography, extrinsic factors such as picture noise, fatigue, abstractions, and human delusion must be addressed, as the rate of breast mass misdiagnosis during early mammography screenings is more than 30%^5^.

Medical image processing algorithms for histopathology pictures are developing quickly, but it is still highly required to provide an automated methodology to achieve effective and highly accurate results. One of the Machine Learning (ML) applications is enhancement in health systems. In classical ML techniques, the dynamic nature of tasks such as pre-processing, feature extraction, etc. decreases the system’s accuracy and efficiency. The idea of DL has been implemented for extracting relevant knowledge from the raw images and using it effectively for classification processes in order to solve the problems of traditional ML techniques^6^.

Without the requirement for manual feature extraction, a convolutional neural network (CNN or ConvNet) model learns directly from the input. It’s powerful technique to detect objects, persons, and scenes by recognizing patterns in photographs. CNNs’ most impressive quality is their remarkable generalizability to different recognition tasks. In particular, CNNs learn to discriminate between different aspects of an input picture, and their architecture is designed to make use of the 2D nature of the image. Large annotated datasets, which are limited in the medical field, particularly for BC, are required to train deep CNNs^7–9^.

**Figure 2:**
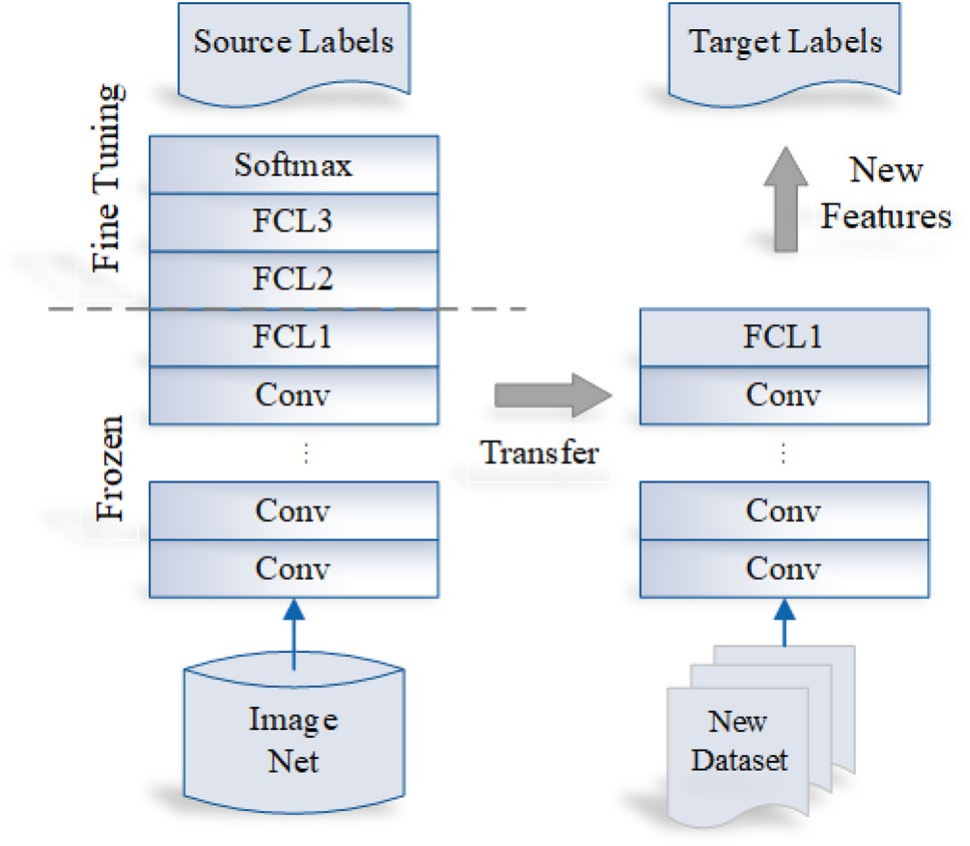
Transfer learning process.

In DL applications, TL is widely used. In the medical sector, it has been very useful, where the amount of data is usually reduced. The aim of TL is to enhance learning by enhancing the target task by transferring information from the source task. Fig. 2 illustrates the TL process.

In this research, a novel DL-based model is created and used for breast tumor detection and classification. There are essentially two parts to the approach provided here. Firstly, five main steps after removing mammographic noise from the dataset (Normalization, segmentation, resizing, sampling, and data augmentation) are utilized to improve the image’s contrast. Next, the pre-trained SueezeNet and DenseNet CNNs are used to transfer the learned parameters to the breast tumor classification task. The full description of INbreast dataset can be founded at kaggle (https://www.kaggle.com/datasets/ramanathansp20/inbreast-dataset?resource=download). The main motivations for this paper have been outlined below:

1. Improving the intensity of the images by applying six phases of data pre-processing.
2. Reducing the training computation time by extracting only the affected regions using segmentation.
3. Overcoming the overfitting problem which occurred due to the lack of medical images.
4. Adapting the SqueezeNet and DenseNet architectures using LSTM and ADAM optimizer to improve the classification performance.
5. Five various measures are applied to test the developed model namely: accuracy, sensitivity, specificity, precision, AUC, and F-score.
6. Detecting and classifying the anomalies in breast mammography more precisely.

The structure of this paper is as follows: the associated work is detailed in “Related works”, whereas “Proposed model”  presents a description of the suggested model for BC detection and classification utilizing TL approaches. “Results”  presents the experimental outcomes compared to the actual data. Finally, the context is concluded in “Conclusion”.

## Related works

Medical specialists sometimes make mistakes when recognizing a condition based on their experience. For the classification of BC, several approaches have been devised. To detect and diagnose the disease, ML and DL algorithms are being applied, with promising results in most situations. They analyze historical data acquired from previous patients to identify relationships between diseases, symptoms, and therapies for the patients.

The diagnosis can be 91.1% more accurate with the use of ML and DL technologies, compared to only 79.9% when performed by a qualified clinician^6^. However, there is still room to create and deploy a more effective BC diagnosis system using an acceptable method.

The innovative CNN for breast tumor classification was developed by Ting et al.^10^. This suggested CNN has only one input layer, twenty-eight hidden layers, and one output layer. The overfitting issue is fixed, and the data set is expanded through the rotation method. By using the BreastNet CNN, Toaçar et al.^11^ were able to extract the most useful information from their breast database. When compared to VGG16, VGG19, and AlexNet, the accuracy achieved by BreastNet (98.80 percent) is superior.

Breast mammography pictures may be classified as benign or malignant using a multi-layer DL model, which was first presented by Abbas^12^. Application to the MIAS dataset yielded values of 92% for sensitivity, 84.20% for specificity, 91.50% for accuracy, and 0.91 for AUC, while Mahmood et al.^13^. developed a novel DL model called ConvNet for diagnosing breast malignancy tissues and achieved an accuracy of 97%, sensitivity of 99%, and an AUC of 0.99.

Breast tumor categorization deep architecture was evaluated by Sha et al.^14^ using the MIAS. To improve the suggested CNN layers, the grasshopper optimization technique was used. In terms of sensitivity, specificity, and accuracy, the improved networks perform at 96%, 93%, and 92%, respectively.

The CNN suggested by Charan et al.^15^ has 13 layers (6 convolutions, 4 average-pooling, and 3 fully connected). Classification is performed using the softmax (SM) function on an input picture with dimensions of $224 \times 224 \times 3. 65\%$ accuracy was achieved when compared to the MIAS database.

In^16^, Wahab et al. used the parameters learned from a pre-trained CNN to classify mitoses. Their approach yielded an accuracy of 0.50 and a recall of 0.80.

Lotter et al.^17^ fine-tuned the ResNet50 model to classify breast tumors. Their model could categorize breast images into five groups. For sensitivity, specificity, and AUC, their model achieved 96.2%, 90.9%, and 0.94, respectively.

The film mammography number 3 (BCDR-F03) dataset was utilized to fine-tune the GoogleNet and AlexNet models by Jiang et al.^18^. GoogleNet achieved 0.88 accuracy, while AlexNet achieved 0.83 accuracy.

In order to fine-tune GoogleNet, VGGNet, and ResNet models, Khan et al.^19^ used a common benchmark dataset. The accuracy of the model was 97.525 percent.

Cao et al.^20^ used random forest dissimilarity to improve TL performance on ResNet-125. After being put through its paces on the “ICIAR 2018” dataset, the model under test showed a classification accuracy of 82.90%.

Deniz et al.^21^ used the AlexNet and VGG16 CNN parameters learned on the BreaKHis mammographic dataset to train a target breast model. Overall, their model was accurate to the tune of 91.37 percent. Celik et al.^22^ used the DenseNet-161 CNN on the same dataset and got 91.57% accuracy.

The breast tumor categorization was enhanced by the application of the learned parameters from VGG-16, VGG-19, and Inception V3 by Abeer et al.^23^. The suggested model is tested on the MIAS dataset. In this study, we demonstrate that the VGG-16 can identify and categorize BC with an overall accuracy of 96.8%.

Abeer et al.^24^ proposed a DL model based on the TL technique. The proposed model contains two major parts. Their model is based on fine-tuned five of the most popular pre-trained CNNs (VGG16, VGG19, Inception-V3, Inception-ResNet, and ResNet-50). The extracted features, except the last three layers, are transferred to train the breast model. The MIAS data are used to train the last layers and to evaluate the presented model. The overall results are 98.96%, 97.83%, 99.13%, 97.35%, 97.66%, and 0.995 for accuracy, sensitivity, specificity, precision, F-score, and AUC.

Abeer et al.^25^ presented a DL methodology depending on the TL technique for breast tumor detection and classification. They used the INbreast dataset to fine-tune the VGG16 and VGG19 networks. The proposed model obtained 97.1%, 96.3%, 97.9%, and 0.988% for accuracy, sensitivity, specificity, and AUC, respectively.

Akselrod-Ballin et al.^26^ evaluated a DL model on the INbreast dataset for breast tumor classification based on a region-based CNN. The presented model achieved an accuracy rate of 78%.

Using GoogleNet, VGGNet, and ResNet, three pre-trained CNN architectures, Khan et al.^19^ constructed a TL model for breast tumor classification. On a common benchmark dataset, this model scored 97.525 percent accurately.

To categorize BC, Al-Antari et al.^27^ created a DL model using feedforward CNN, ResNet 50, and Inception ResNet-V2 networks. The accuracy of the shown model increased from 86.74% to 92.55% to 95.32% throughout the INbreast dataset while using the first, second, and third networks.

Lou et al.^28^ proposed a DL model based on fine-tuning the ResNet-50 pre-trained network for breast tumor classification. The presented model achieved 84.5%, 77.2%, 88.2%, and 0.931 for accuracy, sensitivity, specificity, and AUC, respectively, on the INbreast database.

**Figure 3:**
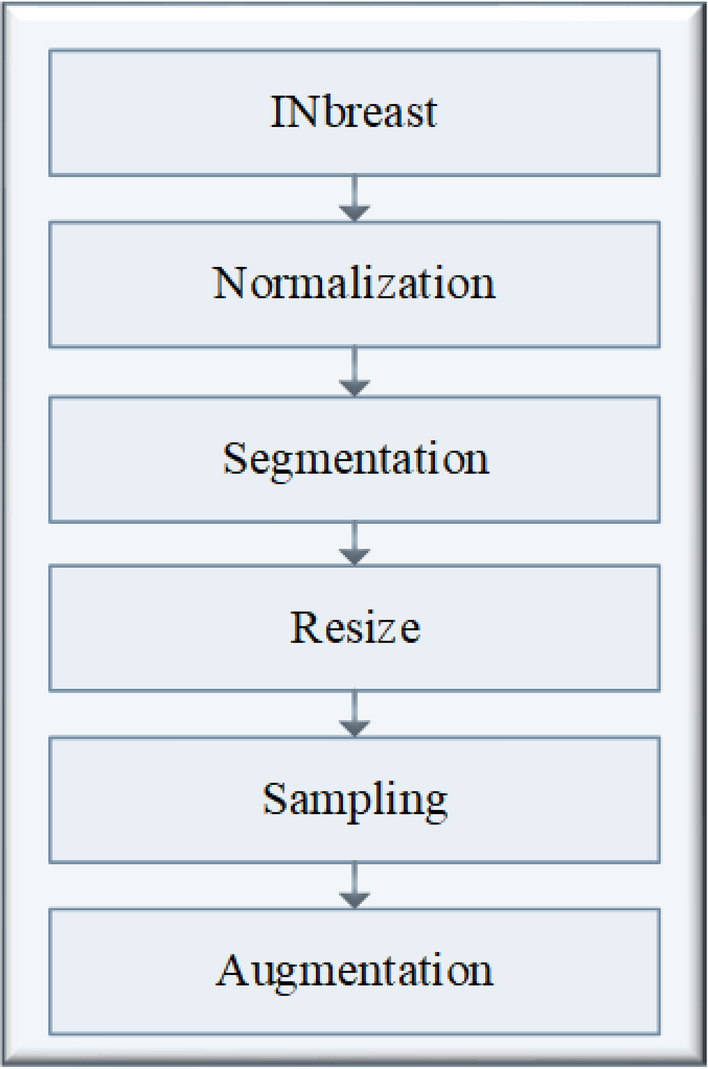
Data pre-processing steps.

El Houby et al.^29^ built a novel CNN to classify breast tumors as benign or malignant. The INbreast, MIAS, and DDSM datasets are used for network evaluation. The CLAHE algorithm is used to improve the image contract. On the INbreast database, the overall results are 96.5%, 96.6%, 96.5%, and 98.0 for accuracy, sensitivity, specificity, and AUC, respectively. Mahmood et al.^5^ implemented a hybrid DL model for improving mass recognition accuracy in mamographic images. First, the features are extracted using a deep CNN network and then used to train an SVM classifier for the best classification accuracy. The proposed model was evaluated using a combination of three breast datasets (MIAS, INbreast, and Private) and achieved an overall accuracy of 97.8%.

Singh et al.^30^ developed and implemented a ML framework for BC classification. The illustrated framework was evaluated using the INbreast dataset and achieved 90.4%, 92.0%, 88.0% for accuracy, sensitivity, and specificity, respectively.

Chakravarthy et al.^31^ presented a DL model based on the TL technique. The presented model transferred the learned parameters except the last three layers from AlexNet, GoogleNet, ResNet-50, and Dense-Net121 and fine-tuned these layers using the INbreast dataset. The support vector machine (SVM) classifier is used to classify the breast tumors classes. The presented model achieved an accuracy of 96.6%.

## Proposed model

### Data pre-processing

**Figure 4:**
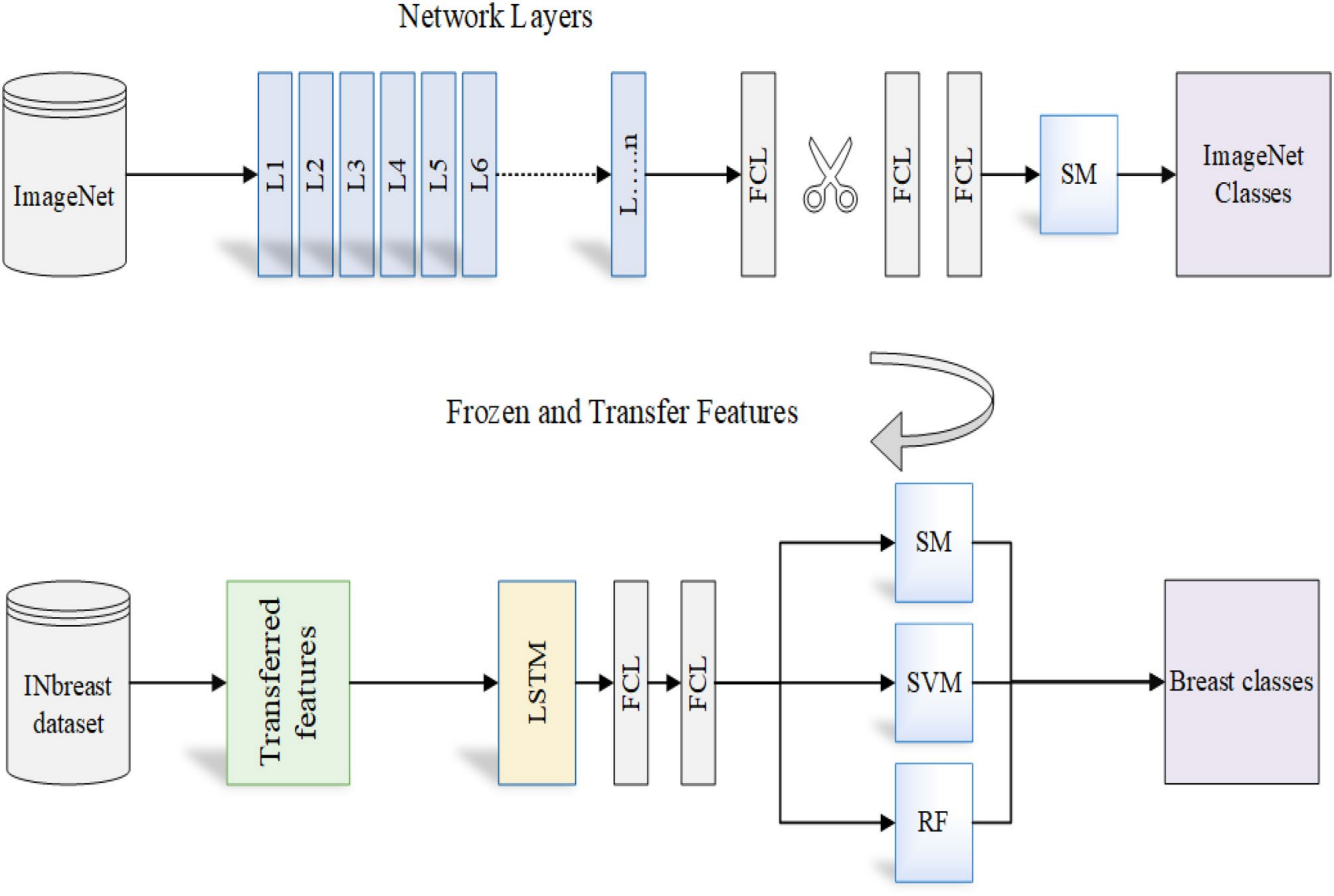
The proposed framework.

Median, Gaussian, and Bilateral filters are applied to remove any mammographic noises from the INbreast dataset. The proposed model in this paper contains two major components, as shown in Figs. 3 and 4. The first is for image preprocessing (IP) while the second is the updated architecture for the deep squeeze CNN for breast feature extraction and classification. The IP contains six phases to improve the image contrast, reduce the computation time, and improve the classification performance, as shown in Fig. 3.

**Figure 5:**
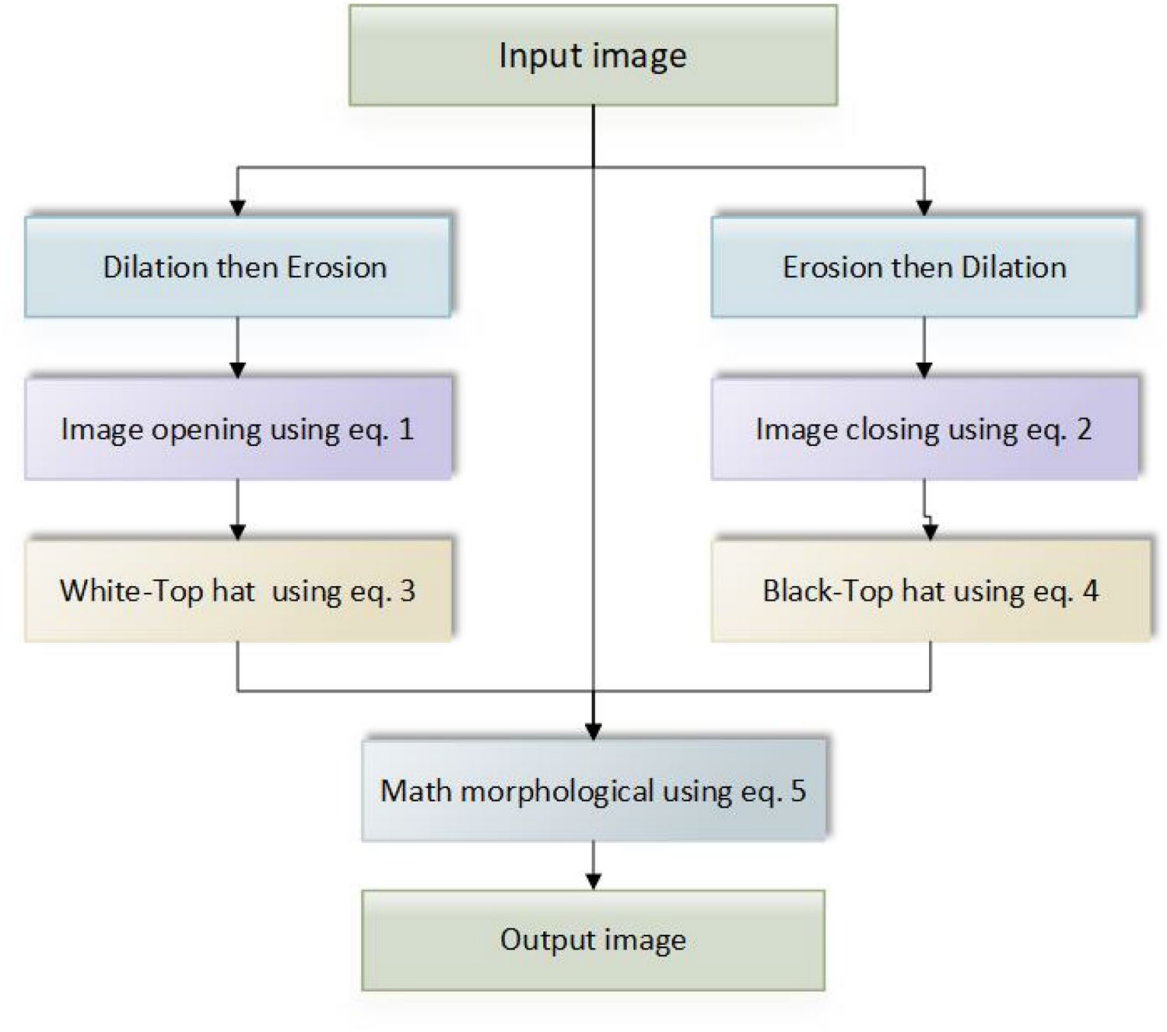
The morphological analysis tasks.

1. Normalization:
  The process of normalizing modifies the range of pixel intensity levels. It is usually called contrast stretching. A group of data should have a consistent dynamic range to prevent mental fatigue or distraction.
2. Morphological analysis:
  The tumor is extracted to reduce the computation time using morphological analysis (MA) and segmentation techniques. The MA process is a very necessary step in which the non-breast regions have been removed using the structuring element (SE).
  The mathematical morphological operation illustrated in Fig. 5. can be estimated as follows:
  - Image opening $$(IM_O)$$
    IM_O is important to remove the small object from the image.
    1 $$\begin{aligned} IM_O=Input \ominus SE \oplus SE \end{aligned}$$
  - Image closing $$(IM_C)$$
    IM_C is useful for filling tiny holes in images while maintaining the shape and size of objects.
    2 $$\begin{aligned} IM\_C=Input \oplus SE \ominus SE \end{aligned}$$
  - White Top-hat (WTH):
    3 $$\begin{aligned} WTH=Input-IM_O \end{aligned}$$
  - Black Top-hat (BTH):
    4 $$\begin{aligned} BTH=IM_C-Input \end{aligned}$$
  - Mathematical morphological $$(M_M)$$:
    5 $$\begin{aligned} M\_M=Input+WTH-BTH \end{aligned}$$
  Where $\ominus$ and $\oplus$ refer to dilation and erosion operations, respectively.
3. Segmentation:
  The tumor tissues have been determined using a region-based segmentation method. Region-based interventions target pixels that share similar characteristics. These methods are simple to use and noise-resistant. Similarity criteria are used in an effective seed-pixel-based region-growing segmentation to assess and add neighboring pixels to a region. The method is repeated until no more pixels meet the requirements.
4. Image resizing:
  The input images are resized to 224 x 224 and translated to three channels to match the input size of the pre-trained Squeeze Net architecture.
5. Data sampling:
  The dataset is divided into 80% and 20% for training and testing, respectively.
6. Data augmentation:
  Data augmentation strategies involve adding slightly modified copies of current data or creating new synthetic data from existing data to enhance the amount of data. It functions as a regularizer and minimizes overfitting, while an ML model is being trained. Data analysis is closely associated with oversampling. Data augmentation is only performed after the data has been divided. For appropriate data augmentation execution, the generated data for training the model must be derived only from the training data.

The data augmentation methods include:

1. Random cropping: Before splitting one image into several images, choose many correct corner points at random. This approach ensures that the cropped images are not duplicated.
2. Rotation: The rotation of an image is based on an angle, such as 45 degrees, and it is repeated repeatedly.
3. Color shifting: This approach adds or subtracts numbers from the red, green, and blue channels (RGB). It may aid in the creation of various color distortions.
4. Flipping: The input images might be flipped vertically or horizontally.
5. Intensity variation: To make the images brighter or darker.
6. Translation: The pixels of the image can be modified with (tx, ty) pixels.

**Figure 6:**
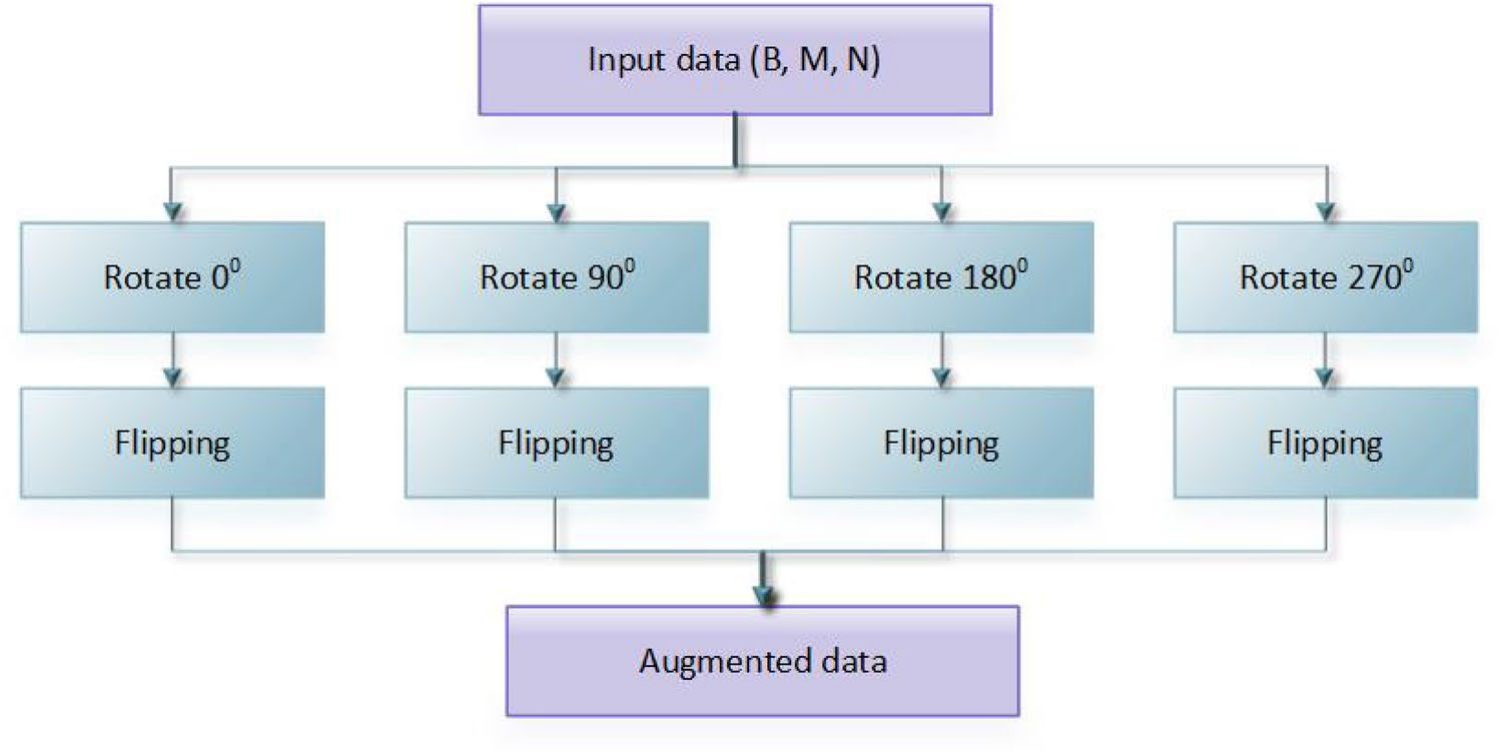
The data augmentation process.


In this paper, the rotation and flipping methods are used to solve the scarcity of the data problem, as shown in Fig. 6.

### Transferring the deep CNN parameters

In this paper, the pre-trained parameters are used to improve the BC classification results. The CNNs contain three main layers (convolution, pooling, and FCL). The squeeze net contains 18 layers and is trained over the ImageNet database. while, the term “Densely Connected Convolutional Networks” is abbreviated as “Densenet.” There is a block in the Densenet where various convolution layers are interconnected. The network complexity increases with each layer, allowing for the identification of larger sections of the images. It begins to identify larger elements of the object until it finally recognizes the desired object. The ImageNet can classify 1000 classes such as pens, trees, cats, dogs, animals, and others.

Next, the SqeezeNet and DenseNet networks are used to detect tumors in breast mammography and classify the detected tumors into benign or malignant using the TL technique. All layers except the last three layers on the squeeze net network are frozen. Then, the frozen layers are transferred, and the last three layers are trained using the pre-processed INbreast images to be able to classify the breast images as illustrated in Fig. 4. However, instead of sending the deep feature sequence directly to the fully connected layer for classification and delivers it to the LSTM layer. The CNN network extracts and recognizes the local and global structures of the image in the pixel series very effectively, whereas the LSTM network discovers long-short-term associations (temporal features). LSTM was utilized to improve the model’s sensitivity and the impact of crucial variables. CNN was implemented to boost the model’s capacity to perceive the sensitivity of the data in the features. As a result, the suggested CNN-LSTM model is better suited for classifying data more precisely.

SqueezeNet is intended to minimise the number of parameters in the network while retaining excellent picture classification accuracy. This is accomplished by combining 1x1 convolutional layers with pooling layers to minimize the spatial dimension of the input feature maps, followed by bigger convolutional layers to capture complicated information. SqueezeNet achieves great accuracy while being more computationally economical than other deep neural network architectures by minimizing the amount of parameters in the network^32^.

**Figure 7:**
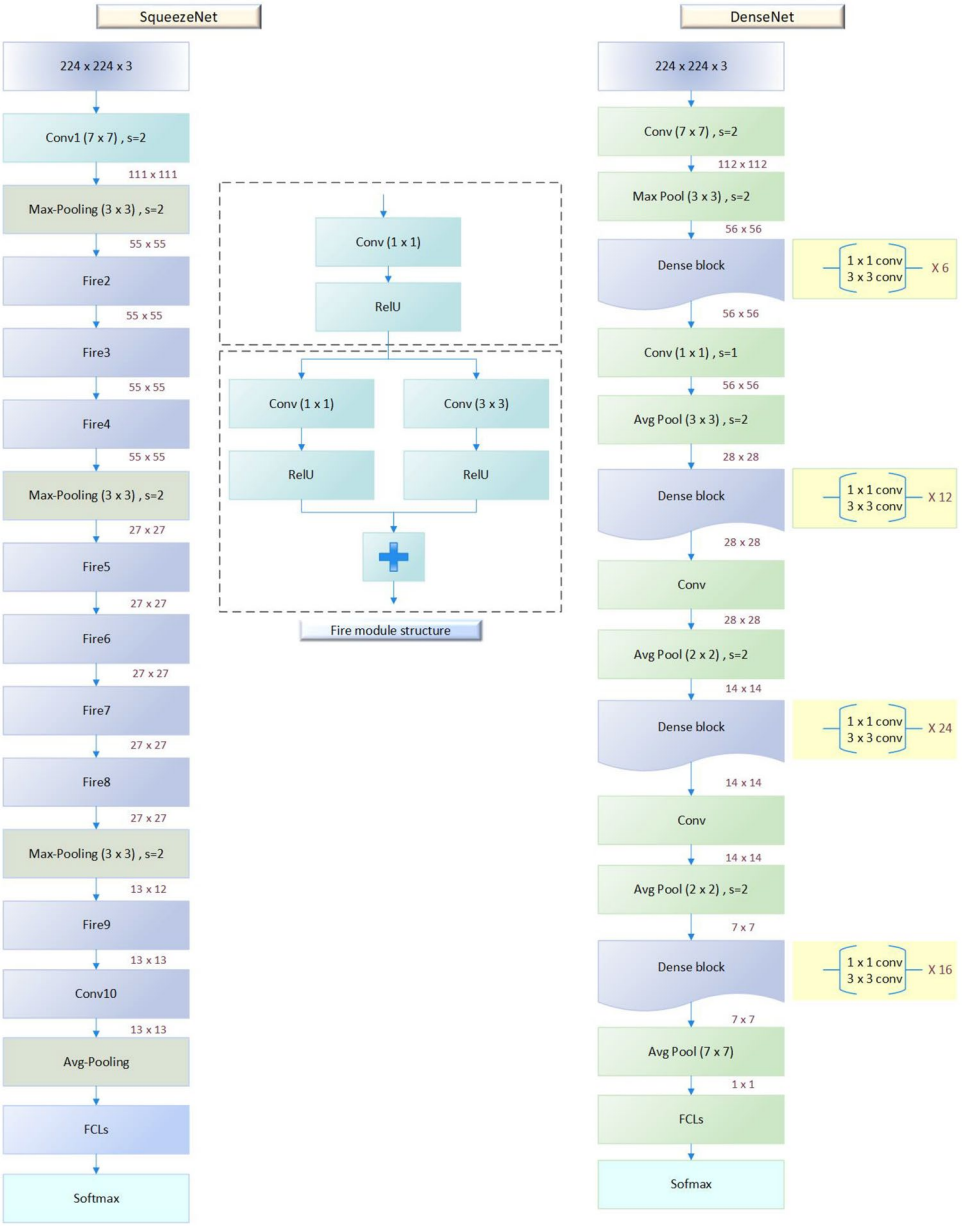
The SqueezeNet architecture.

DenseNet is intended to address the issue of vanishing gradients in very deep neural networks. DenseNet does this by utilizing dense connections between layers, in which the output of each layer is concatenated with the output of all preceding layers in the network. This allows the gradient to flow more readily through the network, making very deep models easier to train. Furthermore, DenseNet has been proven to outperform other deep neural network architectures in terms of accuracy and parameter efficiency, making it a popular choice for image classification applications^33^. The SqueezeNet and DenseNet architectures are illustrated in Fig. 7.

For classification, the SM and multiclass SVM is employed. Using the mathematical formula SM, a vector of numbers is transformed into a vector of probabilities, with the probabilities of each value proportional to the vector’s relative scale. In a multi-class problem, SM assigns a decimal probability to each class. The sum of those decimal probabilities must equal 1.0. Just before the output layer, SM is implemented using a neural network layer. The output layer and the SM layer must both have the same number of nodes. The SM equation is computed, as follows:

6 $$\begin{aligned} \sigma (\vec {V})i=\frac{e^{Vi}}{\sum _{j = 1}^{N}e^{Vj}} \end{aligned}$$

Where $\vec {V}$ is the input vector to the SM function, made up of $(V_0,... V_K),$ *vi* is the elements of the input vector to the SM function $e ^{vi}$ The standard exponential function is applied to each element of the input vector.

$\sum _{j = 1}^{N}e^{Vj}$ is the normalization term. It ensures that all the output values of the function will sum to 1 and each be in the range (0,1).

*N* is the number of classes.

SVM is a supervised ML technique that can be applied to solve problems like classification and regression. Its purpose is to find the most optimal boundary between the various outputs.

The goal of multi-class SVM classification is to map data points into a high-dimensional space so that two classes can be separated linearly. A One-to-One strategy separates the multiclass problem into numerous binary classification problems using this method.

Another strategy is to use the One-to-Rest approach. In this strategy, the breakdown is set to a binary classifier per class. This paper employs the One-to-One strategy, with the classifier employing (m (m − 1)) /2 SVMs, where m is the number of unique class labels.

Stochastic gradient distant with moment (SGDM) and Adam optimizers is used for fine-tuning using the same parameters before and after pre-processing^34,35^. The parameters for the SGDM and Adam optimizers are presented in Tables 1 and 2.

To ensure clarity and facilitate reproducibility, we have provided a detailed step-by-step description of our methods in the form of pseudo code. This can be found in Algorithm 1 of our revised manuscript. The pseudo code outlines the sequence of operations performed during our experimental process, including the initialization of models, data splitting, optimizer definition, model training, addition of an LSTM layer, and classification.

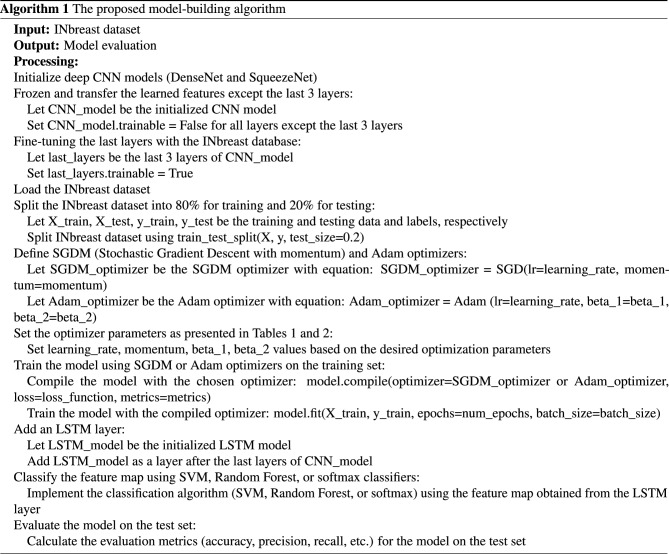

**Table 1:** SGDM parameter settings.

Sr. no	Parameter	Value
1	Training data	80%
2	Testing data	20%
3	Minimum batch size	20
4	Learn-rate drop factor	0.5
5	Maximum Epochs	20
6	Learn-rate drop period	5
7	Initial-learn rate	1e−4

**Table 2:** ADAM parameter settings.

Sr. no	Parameter	Value
1	Training data	80%
2	Testing data	20%
3	lr	0.01
4	$\beta$ 1	0.9
5	$\beta$ 2	0.999
6	Decay	0
7	AMSGrad	False

## Results

### Mammographic dataset

**Figure 8:**
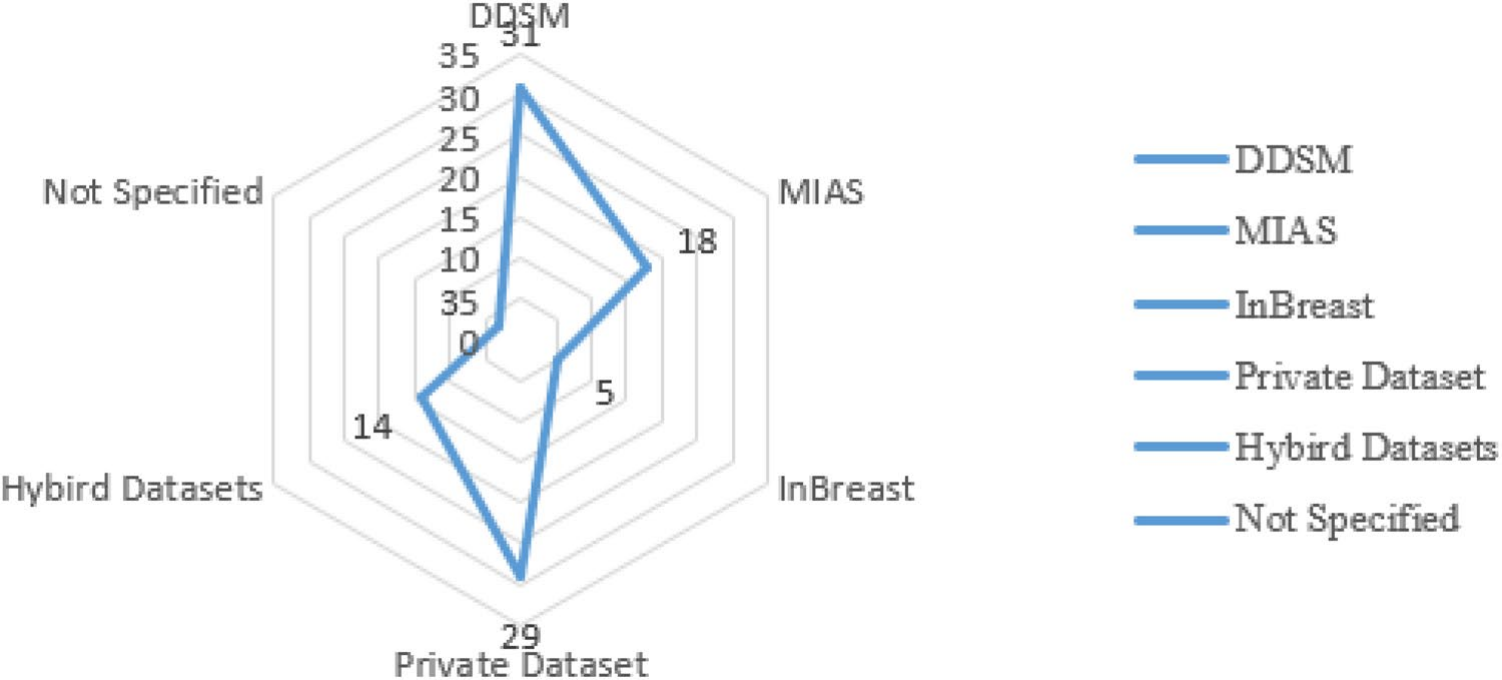
Breast cancer most popular datasets.

In this paper, the INbreast dataset is used to test the proposed model. INbreast is considered one of the most popular datasets in BC, as discussed in^36^. It contains 410 images for six classes in digital imaging and communications in medicine format. The dataset contains not only the image but also some related metadata The Breast Imaging-Reporting and Data System (BI-RADS) value is used to determine the type of tumor. Figure 8, shows the statistics of the most popular dataset usage in BC classification^37^. The detailed information of the INbreast dataset is illustrated in Table 3.

### Experimental results

In this subsection, the experimental results of the suggested approach are presented in details. All experiments have been carried out using a dataset called INbreast. These images have been segmented into 20% for testing and 80% for training, with each class including either benign, malignant, or normal samples. SqueezeNet, as a Transfer-based (TL-based) method, is used in three different scenarios. The first experiment occurs before any pre-processing, the second experiment employs the SM classifier during pre-processing, and the last experiment employs the MSVM classifier.

To be able to have a fair comparison, all three experiments have been performed using Matlab 2021b on Windows 10 on Intel Corei7 machine, 2.67G CPU and 8.00 G of RAM.

The performance was measured using the evaluation metrics for three classes, as shown in Table 4. These metrics are Accuracy (Eq. 7), Sensitivity (Eq. 8), Specificity (Eq. 9), and Precision (Eq. 10).

7 $$\begin{aligned} \text {Accuracy}= & {} \frac{TP}{TP + TN} \end{aligned}$$

8 $$\begin{aligned} \text {Sensitivity}= & {} \frac{TP}{TP + FN} \end{aligned}$$

9 $$\begin{aligned} \text {Specificity}= & {} \frac{TN}{TN + FP} \end{aligned}$$

10 $$\begin{aligned} \text {Precision}= & {} \frac{TP}{TP + FP} \end{aligned}$$

**Figure 9:**
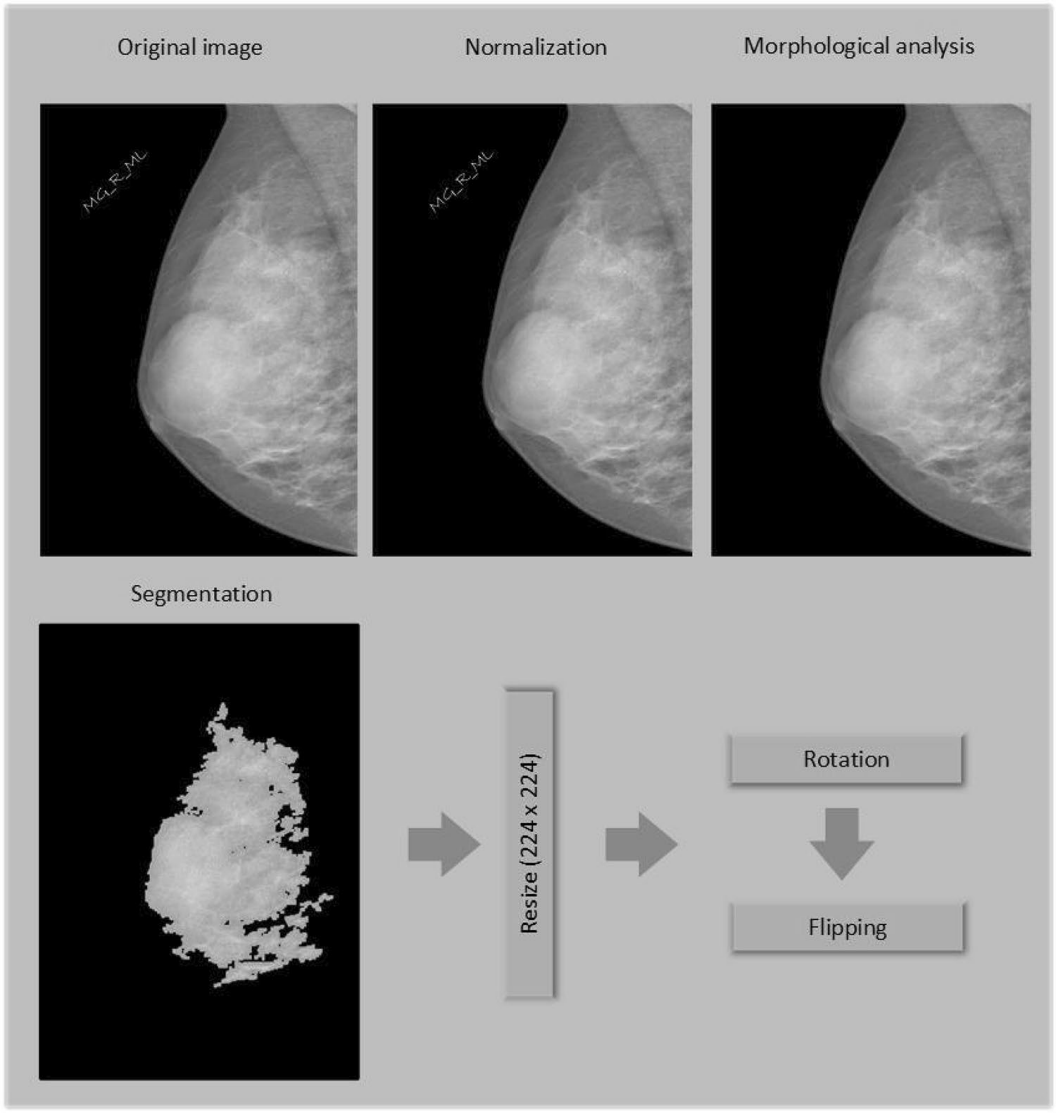
The pre-processing results in INbreast dataset.

Figure 9, shows the breast images after applying the pre-processing phases. The normalization and morphological operations improve the breast image for effective segmentation results while the segmentation results reduce the computation time.

The adapted pre-trained model is applied first using SGDM optimizer and then using the Adam optimizer. Using the SGDM optimizer, The SqueezeNet results before and after pre-processing per class are shown in Table 5. It’s obvious that from the pre mentioned table that using MSVM classifier gets the best results in accuracy, sensitivity, and AUC criteria. on the other hand, the classification results using the RF algorithm achieve the best results in specificity and precision criteria. The same network achieves best results in breast images classification using the ADAM optimizer as shown in Table 6. The results shown that the MSVM classifier is achieved the best results in almost all evaluation criteria as its results reached to 99.2%, 98.8%, 99.1%, 96%, and 0.998 for accuracy, sensitivity, specificity, precision, and AUC, respectively.

The results using the DenseNet architecture and SGDM optimizer before and after pre-processing are presented in Tables 7. The results from this table is shown that the MSVM classifier is achieved the best results in accuracy, sensitivity, precision, and AUC with 95.9%, 95.9%, 90.6%, and 0.998, respectively.While, the best specificity result is achieved using the RF classifier with 90.8%. The same network achieves the best results in breast images classification using the ADAM optimizer as shown in Table 8, its observed that the overall results are proved that the network optimized using the Adam optimizer is more accurate than the network optimized using the SGDM optimizer . The performance is compared with three other existing models. It can conclude that MSVM archives better results from SM and RF classifier in Adam in almost criteria i. e. accuracy, sensitivity, specificity, and AUC with 97.33%, 98%, 95.6%, and 99.8%, respectively.But, the best precision result is achieved using the RF classifier. The results analysis prove that the presented model performs better than other existing models in terms of accuracy, sensitivity, specificity, and precision.

Moreover, a comparison with different state-of-art approaches have been done with our proposed model as seen in Table 9. From this table, it can be seen that the developed model has better results comparing to recent studies existed in literature in terms of accuracy, sensitivity, specificity, precision, and AUC.

**Table 3:** Dataset description.

BI-RADS value	Class	Number
1	Normal	67
2	Benign	220
3	Benign	23
4	Malignant	44
5	Malignant	49
6	Malignant	8

**Table 4:** Three classes of confusion matrices.

Class		Predict		
		Benign	Malignant	Normal
Actual	Benign	$TP_B$	$EM_B$	$EN_B$
	Malignant	$EB_M$	$TP_M$	$EN_M$
	Normal	$EB_N$	$EM_N$	$TP_N$

**Table 5:** The results applied using the SqueezeNet pre-trained CNN and SGDM optimizer per class.

CNN	Class	Performance of the Classifier				
		Accuracy (%)	Sensitivity	Specificity	Precision	AUC
Before pre-processing	Benign	64.19	0.35	0.66	0.39	0.44
	Malignant	59.82	0.38	0.62	0.369	0.45
	Normal	61.63	0.29	0.68	0.332	0.44
	Average	61.88	0.34	0.65	0.36	0.44
After pre-processing	Benign	96.82	0.90	0.99	0.941	0.99
(SM)	Malignant	97.1	0.957	0.97	0.81	0.987
	Normal	95.3	0.96	0.932	0.95	0.981
	Average	96.4	0.939	0.964	0.90	0.986
After pre-processing	Benign	98.36	0.1	0.98	0.91	0.994
(MSVM)	Malignant	97.9	0.96	0.981	0.923	0.998
	Normal	98.45	0.965	0.99	0.931	0.997
	Average	98.236	0.975	0.983	0.921	0.996
After pre-processing	Benign	99.1	0.98	0.985	0.92	0.997
(RF)	Malignant	97.3	0.958	0.99	0.92	0.996
	Normal	98.1	0.97	0.987	0.927	0.997
	Average	98.16	0.969	0.987	0.922	0.996

**Table 6:** The results applied using the SqueezeNet pre-trained CNN and ADAM optimizer per class.

CNN	Class	Performance of the Classifier				
		Accuracy (%)	Sensitivity	Specificity	Precision	AUC
Before pre-processing	Benign	63.9	0.37	0.69	0.41	0.45
	Malignant	60.2	0.35	0.65	0.38	0.46
	Normal	64.2	0.34	0.68	0.37	0.44
	Average	62.76	0.353	0.673	0.386	0.45
After pre-processing	Benign	97.1	0.89	0.99	0.95	0.998
(SM)	Malignant	98.2	0.949	0.979	0.84	0.998
	Normal	95.9	0.97	0.94	0.95	0.995
	Average	97.06	0.936	0.969	0.91	0.997
After pre-processing	Benign	99.5	1.0	0.988	0.93	0.998
(MSVM)	Malignant	98.9	0.987	0.99	0.95	0.999
	Normal	99.2	0.978	0.995	1.0	0.998
	Average	99.2	0.988	0.991	0.96	0.998
After pre-processing	Benign	99.2	0.99	0.99	0.94	0.991
(RF)	Malignant	99	0.979	0.987	0.947	0.998
	Normal	98.4	0.98	0.992	0.99	0.998
	Average	98.86	0.983	0.989	0.959	0.995

**Table 7:** The results applied using DenseNet and SGDM optimizer per class.

CNN	Class	Performance of the Classifier				
		Accuracy (%)	Sensitivity	Specificity	Precision	AUC
Before pre-processing	Benign	56.54	0.31	0.56	0.35	0.41
	Malignant	51.82	0.32	0.52	0.34	0.45
	Normal	54.63	0.22	0.68	0.33	0.43
	Average	54.33	0.28	0.586	0.33	0.43
After pre-processing	Benign	94.7	0.91	0.93	0.92	0.981
(SM)	Malignant	93.92	0.94	0.96	0.88	0.98
	Normal	94.9	0.93	0.94	0.90	0.99
	Average	94.5	0.926	0.943	0.90	0.983
After pre-processing	Benign	96.1	0.96	0.95	0.88	0.999
(MSVM)	Malignant	95.5	0.958	0.97	0.90	0.998
	Normal	96.2	0.96	0.94	0.94	0.997
	Average	95.9	0.959	0.953	0.906	0.998
	Benign	97.2	0.97	0.94	0.89	0.999
(RF)	Malignant	95.3	0.96	0.97	0.904	0.998
	Normal	95.1	0.94	0.96	0.93	0.998
	Average	95.86	0.956	0.95	0.908	0.998

**Table 8:** The results applied using the DenseNet and Adam optimizer per class.

CNN	Class	Performance of the Classifier				
		Accuracy (%)	Sensitivity	Specificity	Precision	AUC
Before pre-processing	Benign	59.76	0.35	0.57	0.33	0.42
	Malignant	54.2	0.33	0.54	0.35	0.46
	Normal	52.72	0.31	0.69	0.39	0.44
	Average	55.56	0.33	0.61	0.356	0.44
After pre-processing	Benign	95.8	0.93	0.93	0.91	0.99
(SM)	Malignant	94.1	0.92	0.94	0.90	0.98
	Normal	96.1	0.95	0.96	0.90	0.99
	Average	95.3	0.93	0.94	0.903	0.986
After pre-processing	Benign	97.3	0.99	0.96	0.90	0.999
(MSVM)	Malignant	96.9	0.981	0.96	0.91	0.997
	Normal	97.8	0.972	0.95	0.93	0.998
	Average	97.33	0.98	0.956	0.913	0.998
After pre-processing	Benign	98.2	0.98	0.95	0.91	0.999
(RF)	Malignant	96.3	0.97	0.96	0.92	0.998
	Normal	96.9	0.96	0.96	0.939	0.998
	Average	97.13	0.97	0.956	0.923	0.998

**Table 9:** Comparison between existing methods and proposed model.

Method	Accuracy (%)	Sensitivity (%)	Specificity (%)	Precision (%)	AUC
Akselrod-Ballin^26^	78	–	–	–	–
Al-Antari et al.^27^	95.3	–	–	–	–
Meng Lou et al.^28^	84.5	77.2	88.2	–	0.931
El Houby et al.^29^	96.5	96.6	96.5	–	0.98
Singh, H. et al.^30^	90.4	92	88	–	–
Sannasi et al.^31^	96.6	–	–	–	–
Saber et al.^4^	97.1	96.3	97.9	–	0.988
**Proposed**	**99.236**	**98.8**	**99.1**	**96**	**0.998**

Best results are in bold.

## Conclusion

A DL model for breast tumor detection and classification in breast mammography was proposed in this paper. The goal of this model is to assist medical physicians in the detection and diagnosis of BC. The INbreast data were classified into three categories: benign, malignant, and normal. First, the mammographic dataset is preprocessed to improve the intensity of the images and reduce the computation time. Then, the data are increasingly augmented using data augmentation techniques. In the second phase, the SqueezeNet and DenseNet models are applied to enhance the breast features that are extracted from the input images. Finally, the SM, MSVM, and RF classifiers are employed for data classification. The experimental results using the MSVM classifier and Adam optimizer achieved the best results with accuracy, sensitivity, specificity, precision, and AUC values of 99.236%, 98.8%, 99.1%, 96%, and 0.998, respectively.

In future work, the Optimization algorithms can be used to enhance the developed algorithm^38–51^. For example, we can use Snake Optimizer^52^, Fick’s Law Algorithm (FLA)^53^, Dwarf Mongoose Optimization Algorithm (DMOA)^54^, Reptile Search Algorithm (RSA)^55^, Dandelion Optimizer^56^, and Aquila Optimizer (AO)^57^.

## Data Availability

Data is available from the authors upon reasonable request from crospending author. Data is available from the authors upon reasonable request.
